# Evaluating the Efficacy of Inhaled Colistin via Two Nebulizer Types in Ventilator-Associated Pneumonia: Prospective Randomized Trial

**DOI:** 10.3390/antibiotics13111099

**Published:** 2024-11-19

**Authors:** Chung-Chi Huang, Tien-Pei Fang, Chieh-Mo Lin, Chien-Ming Chu, Hsuan-Ling Hsiao, Jui-Fang Liu, Hsin-Hsien Li, Li-Chung Chiu, Kuo-Chin Kao, Chin-Hsi Kuo, Shaw-Woei Leu, Hui-Ling Lin

**Affiliations:** 1 Division of Pulmonary and Critical Care Medicine, Linkou Chang Gung Memorial Hospital, Taoyuan 33342, Taiwan; 2 Department of Respiratory Therapy, Chang Gung University, Taoyuan 33308, Taiwan; 3 Department of Respiratory Therapy, Chiayi Chang Gung Memorial Hospital, Chiayi 61363, Taiwan; 4 Department of Respiratory Care, Chang Gung University of Science and Technology, Chiayi 61363, Taiwan; 5 Division of Pulmonary and Critical Care Medicine, Chiayi Chang Gung Memorial Hospital, Chiayi 61363, Taiwan; 6 Division of Pulmonary and Critical Care Medicine, Keelung Chang Gung Memorial Hospital, Keelung 20401, Taiwan; 7 Department of Pharmacy, Linkou Chang Gung Memorial Hospital, Taoyuan 33342, Taiwan

**Keywords:** nebulizers, anionic, colistin, ventilator associated pneumonia, clinical pulmonary infection score

## Abstract

**Backgroud:** This prospective randomized trial evaluated the clinical efficacy of inhaled colistin administered through two distinct nebulizer types, a vibrating mesh nebulizer (VMN) and a jet nebulizer (JN), in the treatment of ventilator-associated pneumonia caused by multidrug-resistant bacteria. In addition, an in vitro model was used to determine the optimal delivery of colistin. **Method:** Thirty-two patients prescribed intravenous (IV) colistin inhalation were randomized to receive either a VMN (n = 17) or a JN (n = 15), then compared to the control group (IV alone) over a 7-to 10-day period. The primary endpoint was the clinical pulmonary infection score (CPIS), and the secondary endpoints were the Sequential Organ Failure Assessment (SOFA) score, Acute Physiology and Chronic Health Evaluation (APACE) score, and duration of ventilator use. **Results:** Results from in vitro testing demonstrated that VMN delivered a significantly higher colistin dose than JN (35.68 ± 3.55% vs. 23.56 ± 3.31%; *p* < 0.001) when positioned at the humidifier inlet. Compared to the IV alone group, the IV with inhalation group yielded significant improvements in CPIS, SOFA score, and APACHE score on day 7; nevertheless, clinical outcomes between the two nebulizers were statistically indistinguishable. **Conclusions:** In conclusion, although VMN delivers a higher dose in vitro, both nebulizers yielded comparable clinical outcomes. This study was registered at US Clinical Trial Registration (NCT04633317).

## 1. Introduction

The identification of 12 antibiotic-resistant priority pathogens by the World Health Organization indicates an urgent need for innovative treatment strategies. Carbapenem-resistant *Pseudomonas aeruginosa* and *Acinetobacter baumannii* are formidable adversaries [1]. Recent systemic reviews have shed light on the alarming 13% incidence of carbapenem-resistant gram-negative bacteria, underscoring the need to confront these resistant strains [2,3]. In the challenging landscape of ventilator-associated pneumonia (VAP), *P. aeruginosa* and *A. baumannii* pose significant hurdles, complicating clinical management and demanding novel therapeutic approaches that can effectively combat these resilient pathogens [4].

Inhaled antibiotics have emerged as promising avenues for VAP treatment. This delivery method offers the advantage of directly administering concentrated drug doses to the lungs, surpassing the minimum inhibitory concentrations required for effective treatment within the epithelial lining fluid while minimizing systemic exposure [5,6]. The clinical benefits of inhaled antibiotics in VAP treatment are manifold, including the early eradication of pathogens, hastened clinical recovery, and, notably, the absence of any discernible increase in ICU mortality or nephrotoxicity risk [6].

Previous studies by Palmer et al. have provided compelling evidence of the efficacy of inhaled antibiotics in patients with ventilator-associated tracheobronchitis [7,8]. Additionally, previous studies have yielded tangible results, demonstrating reductions in bacterial resistance, lowered clinical pulmonary infection scores (CPIS), and shortened durations of ventilator support [9,10,11]. The revelation from an international survey of inhaled colistin as the predominant antimicrobial prescribed in ICUs underscores its pivotal role in contemporary clinical practice and emphasizes the urgency of optimizing its delivery mechanisms [12]. Recently, a large trial by Erhmann et al. reported that the early administration of inhaled amikacin for 3 days to patients receiving mechanical ventilation for at least 3 days reduced the occurrence of VAP and infection-related ventilator-associated complications [13].

Colistin, typically available in dry powder form, is dissolved in saline and administered intravenously or as an inhalation solution via a nebulizer. The array of nebulizers includes a jet nebulizer (JN) and vibrating-mesh nebulizers (VMNs), with the former being the most widely used nebulizer for mechanically ventilated patients [14]. The selection of a jet nebulizer for clinical use depends on the availability and cost, while a JN is more accessible than a VMN, which is expensive. An international survey reported that approximately 50% of cases use JN vs. 10–15% which use VMN [12]. However, the effectiveness of nebulized drug delivery within a mechanical ventilator system depends on several critical factors, including nebulizer performance and placement, drug viscosity, and ambient humidity levels within the ventilator circuit [15,16]. Vibrating mesh nebulizers have advantages such as minimal residual doses and enhanced peripheral drug deposition compared to jet nebulizers [17]. The aerosol output rate can vary significantly and is influenced by drug formulation properties, such as high viscosity, which may impede aerosol generation [16]. Additionally, the patency of the mesh pores is critical for consistent performance and dependent on appropriate maintenance and cleaning practices [18]. While most studies on inhaled antibiotics have focused on clinical outcomes, the use of delivery devices has often not been well reported [19]. Given the paramount importance of optimizing the delivery of nebulized colistin within mechanical ventilator systems, the comparative advantages of various nebulizer types warrant thorough examination.

In light of these considerations, a prospective clinical trial was designed to explore the efficacy of inhaled colistin administered via two distinct nebulizers against those receiving intravenous colistin, thus providing the comparative efficacy of these delivery modalities in managing ventilator-associated diseases.

## 2. Results

### 2.1. Clinical Outcomes

**Figure 1:**
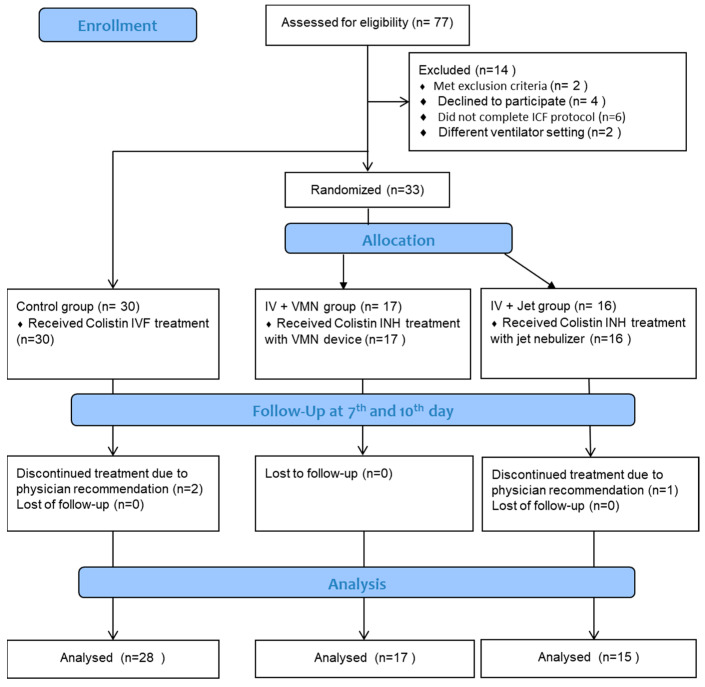
The study flow diagram.

The flow diagram of this study is shown in Figure 1. Seventy-seven patients were initially evaluated, and fourteen were subsequently excluded. A total of 33 patients who received intravenous (IV) colistin were designated as the control group, whereas 33 patients who were prescribed inhaled colistin were randomly allocated to the JN and VMN groups. Sixty patients were included in the final analysis.

The demographic and clinical characteristics of the enrolled patients are summarized in Table 1. The data indicated similarities in patient characteristics among the groups, with most patients exhibiting infection-related symptoms associated with pulmonary diseases or cancers upon hospital admission. In contrast, the other indices showed no significant differences among the groups. Additionally, laboratory parameters, including C-reactive protein (CRP) and white blood cell (WBC) counts, exceeded normal ranges on day 1, indicating the presence of systemic inflammation. Additionally, the renal function parameters are represented by the following.

Creatinine and blood urea nitrogen (BUN) levels were within normal limits before colistin treatment initiation. Oxygenation status, as reflected by the PaO_2_/FiO_2_ ratio and alveolar–arterial oxygen gradient (AaDO_2_), indicated moderate hypoxemia across the patient cohort.

Table 2 compares the primary outcome, CPIS, among the three groups on days 7 and 10 after colistin treatment. The CPIS was significantly higher in the IV group on day 7 (*p* = 0.03), although this difference was not significant on day 10 (*p* = 0.55). The CPIS score was reduced by 1 point in the IV + VMN vs. 1.5 points in the IV + JN group, without statistical significance at the two checking points (*p* = 0.21 and *p* = 0.54).

The SOFA score was significantly higher in the IV group than in both the IV + VMN and IV + JN groups on day 7 (Table 3). Additionally, reductions in the APACHE scores were observed in the inhalation groups on day 7. Interestingly, the median CRP level rose from the baseline on day 7 and then reached normal levels on day 10. Nevertheless, the decreases in APACHE and CRP levels were similar between the nebulizer groups. Furthermore, no statistical differences were observed in primary and secondary outcomes between the IV + VMN and IV +JN groups regarding the delivery by the two nebulizers.

**Figure 2:**
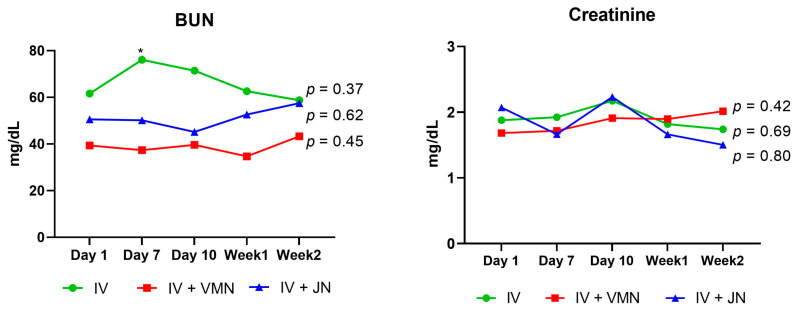
Trends in blood urea nitrogen (BUN) and creatinine levels for nephrotoxicity monitoring. Data are presented as mean. Comparisons among three groups at five time points were analyzed by Freidman test, * *p* < 0.05; comparisons among three groups were analyzed by Kruskal–Wallis test. Abbreviations: IV, intravenous; JN, jet nebulizer; VMN, vibrating mesh nebulizer.

Nephrotoxicity was evaluated using BUN and creatinine measurements on days 7 and 10, as well as 1 and 2 weeks after the administration of inhaled colistin. BUN levels were significantly higher in the IV group than in both inhalation groups on day 7, and decreased on day 10. Comparisons of individual changes over time were analyzed using the Friedman test (Figure 2). Minimal variations were found throughout the treatment period and post-regimen.

Table 4 shows the long-term clinical outcomes. The durations of ICU stays were similar among the three groups, with medians ranging from 30.5 to 36 days (*p* = 0.23), while the hospital stays were shorter in the IV control group without significance (*p* = 0.28). ICU and hospital survival rates were higher in the IV + VMN group, but the difference was not statistically significant (*p* = 0.06).

### 2.2. In Vitro Drug Delivery Testing

**Figure 3:**
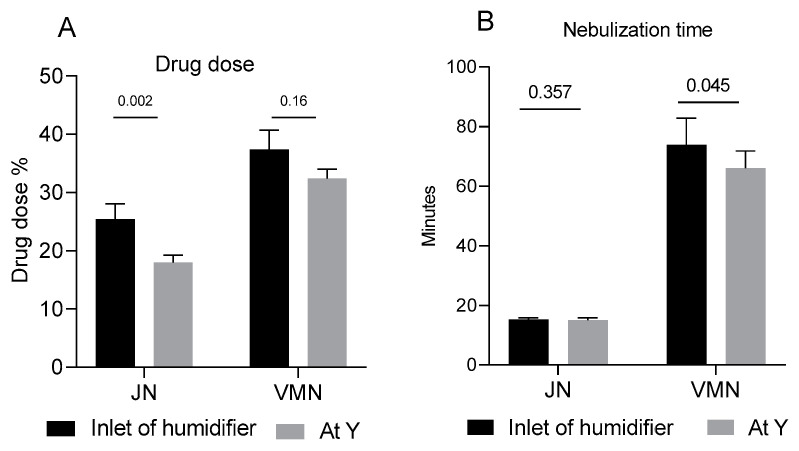
Comparisons of drug dose (% ±SD, (**A**)) collected distal to the endotracheal tube and time (**B**) of delivery using two nebulizers placed at the inlet of the humidifier and between the inspiratory limb and circuit Y.

The in vitro drug delivery test results showed distinct differences between the two nebulizers under investigation. Figure 3A compares the performance of the nebulizer at different placements within the in vitro setup. When positioned at the inlet of the humidifier, the VMN delivered a significantly higher colistin dose than JN (35.68 ± 3.55% vs. 23.56 ± 33.31%, respectively; *p* < 0.0001). Similarly, at the Y-piece of the inspiratory limb, VMN outperformed JN in colistin delivery (32.28 ± 7.48% vs. 21.71 ± 4.40%, respectively; *p* < 0.001). Furthermore, the nebulization time was observed to be significantly longer with the VMN compared to the JN (Figure 3B, 70.0 ± 7.2 min vs. 15.2 ± 0.7 min, respectively; *p* < 0.001), with placement at the heated humidifier resulting in a significantly longer nebulization time compared to placement at the Y-piece of the inspiratory limb (*p* = 0.04).

## 3. Discussion

This study evaluated the comparative efficacy of IV + inhalation colistin delivered by different nebulizer types in patients with VAP caused by multidrug-resistant gram-negative bacteria. While treatment in the IV + inhalation group demonstrated superior efficacy regarding infection status and disease severity compared to the IV-only group, no significant difference was observed between the two nebulizer groups. Efficacy was evidenced by reductions in the CPIS, APACHE scores, and CRP levels in the IV + inhalation group. There were no significant differences in long-term outcomes such as ICU and hospital length of stay or ICU and hospital survival rates.

### 3.1. Clinical Outcomes

When inhaled, nebulized colistin passes through the alveolar epithelial membrane into the epithelial lining fluid, further disrupting the cell membrane and lipopolysaccharide permeability of gram-negative bacilli and achieving an antibacterial effect [20]. Additionally, colistin is a concentration-dependent antibiotic, indicating that its antibiotic effect and bactericidal efficacy depend on achieving sufficient concentration levels. Intravenous administration may lead to low colistin concentrations in plasma and lung tissues, increasing the risk of treatment failure [21]. Therefore, when high-dose inhaled colistin is used, the concentration in lung tissue within the regions affected by bronchopneumonia exceeds the minimum inhibitory concentration by more than five times, approaching the minimum inhibitory concentration breakpoint.

Kyriakoudi et al. investigated the pharmacokinetic characteristics of inhaled colistin using JN and VMN and reported comparable epithelial lining fluids [22]. The epithelial lining fluid colistin concentration was similar in both groups at 1 h and achieved a predefined breakpoint (2 mg/L) at 4 h. Additionally, Lee et al. used a JN to deliver costistin and pharmacokinetically compared the median concentration of colistin in the epithelial lining fluid, which was hundreds of times higher than in plasma, and the minimum inhibitory concentration exceeded 2 mg/L at 6 h [23].

Despite the higher delivered dose with a VMN in our bench study, the clinical results revealed comparable clinical outcomes when a JN or VMN was used to administer colistin. Based on Kyriakoudi’s study and our results, it can be assumed that both types of nebulizers delivered colistin to the lungs and may have achieved the minimal inhibition concentration, resulting in comparable clinical outcomes. Further large clinical trials on the minimum dose of each type of antibiotic delivered by a nebulizer are warranted to confirm this finding.

The CPIS serves as a widely accepted primary outcome measure for evaluating the efficacy of inhaled antibiotics in VAP treatment [9,24]. Previous studies have consistently demonstrated improvements in CPIS scores with adjunct inhaled colistin therapy [10]. Similarly, our results align with these findings, showing significantly lower CPIS scores in the inhalation groups on day 7 despite no significant differences in the baseline CPIS. The IV-alone regimen appeared to have a postponed effect, showing reduced CIPS scores on day 10. vs. both inhalation groups on day 7.

Additionally, reductions in APACHE scores were observed in the inhalation groups on day 7, further supporting the efficacy of adjunct inhaled colistin in improving the clinical outcomes in patients with VAP. The long-term clinical outcomes were insignificant despite the significant improvements in CPIS, SOFA score, APACHE score, CRP levels, and BUN levels in the IV + inhaled groups. However, adjunct inhaled colistin did not significantly affect mortality rates, which aligns with the findings of our study.

### 3.2. Nephotoxicuty and Complications

The occurrence of nephrotoxicity in patients treated with polymyxins is associated with the type of drug, dose, age, pre-diagnosed renal disease, diabetes mellitus, and vasopressor use [25]. Previous studies have reported that drug-related nephrotoxicity caused by colistin accounts for 14–26% of all acute kidney injury cases in the adult population [26]. Moreover, a meta-analysis demonstrated that adjunct inhaled colistin use is associated with an increased risk of bronchospasm, while no significant association with neurotoxicity was observed [27]. Nephrotoxicity is generally defined as a serum creatinine level increase of ≥1.5 mg/dL within 7 days. This study adopted this universal definition of tail termination [28]. We predefined nephrotoxicity when reducing creatinine clearance; however, none of the participants reported increased creatinine clearance. Our results reaffirm the safety profile of nebulized colistin therapy.

Interestingly, our results showed that BUN in the IV alone group significantly increased from 10.6 to 64.3 mg/dL on day 7 and remained as high as 56.6 mg/dL on day 10. Compared to the changes in the two inhalation groups, BUN levels remained similar in the groups of IV + JN, in contrast to a reduction from 31.3 mg/dL to 19.3 mg/dL with the IV + VMN on day 7. An increased BUN level is often used as a biomarker for renal failure. However, increased BUN levels are also associated with several conditions, such as older age and heart failure. Drug-induced nephrotoxicity changes glomerular hemodynamics, tubular cell toxicity, inflammation, and crystal nephropathy. Acute tubular damage of a necrotic nature at the level of proximal tubule cells is considered the primary event in colistin-induced kidney injury, which can be monitored according to creatinine level and urine output [26,29]. The alteration of BUN levels may be independent of colistin administration.

Inhaled antibiotics have been found to increase airway complications, especially in patients with hypoxemia. A high drug concentration may induce tracheobronchitis [30]. The potential adverse effects include cough, bronchospasm, hypersensitive pneumonitis, and hemoptysis [31,32]. Sahakijpijar et al. calculated the correlation between cough incidence and aerosolized drug dosage. The incidence of cough increased when the drug dose exceeded 30 mg [32]. Our results revealed no incidence of pulmonary complications when 66.8 mg of colistin was administered.

### 3.3. Selection of Nebulizers

Upon selecting an aerosol device for the administration of inhaled antibiotics, an international survey reported that JN was the most commonly used, possibly because of the cost of the nebulizers, regardless of in vitro models, indicating a higher delivery dose of aerosolized drugs with a VMN than the others [12,17]. The cost would be significantly higher with a VMN than a JN with the same treatment dosage. This is primarily due to the higher initial cost of the VMN, despite its efficiency advantages. Jet nebulizers cost approximately USD 1, whereas VMNs are more expensive at around USD 100 in our region. The substantial cost difference could indeed be prohibitive in resource-limited settings, particularly in developing countries where healthcare budgets and patient affordability may be constrained.

This was the first study to compare the clinical outcomes of inhaled colistin administered via two different types of nebulizers. While our in vitro studies have favored VMN over JN for nebulized antibiotic delivery due to the lower residual dose and higher emitted dose, our clinical study did not show a significant difference in outcomes between the two nebulizer types. This disparity highlights the complexity of translating in vitro findings to clinical practice. It underscores the need for larger clinical trials to comprehensively investigate the correlation between inhaled dose, alveolar epithelial lining fluid concentration, minimal effectiveness dose, and therapeutic effect. This suggests that factors beyond the delivered dose, such as patient-specific characteristics or variations in nebulizer performance in clinical settings, may influence the therapeutic efficacy. Consequently, further studies exploring the optimal colistin doses for different nebulizer types are warranted to inform treatment decisions and optimize therapeutic outcomes for VAP management. By leveraging a comprehensive understanding of nebulizer technology, drug delivery mechanisms, and patient-specific factors, clinicians can tailor treatment strategies to maximize therapeutic efficacy and minimize the adverse effects in patients with VAP.

### 3.4. Limitations

The limitations of this study must be acknowledged. Although notable, discrepancies in outcomes between the nebulizer groups did not reach statistical significance, possibly due to the limited sample size. The required sample size was 30 participants per group. Informed consent could not be obtained owing to limited family visits to the ICUs during the COVID-19 pandemic. Additionally, aerosol therapy was discouraged to prevent air transmission. Furthermore, the study protocol allowed colistin therapy for up to 10 days, but some patients required a further colistin regimen after the experimental period, potentially impacting long-term outcomes. These limitations underscore the need for larger, more comprehensive studies to validate and further elucidate the findings of this study, ultimately informing clinical practice and optimizing the treatment strategies for patients with VAP.

## 4. Materials and Methods

### 4.1. Clinical Outcome Measurements

This open-label, prospective clinical trial was conducted from December 2020 to July 2022 across multiple Chang Gung Memorial Hospital branches, including Keelung, Linkou, and Chiayi in Taiwan. Ethical approval for the study was obtained from the Chang Gung Memorial Foundation Ethics Committee, and the trial was registered on ClinicalTrials.gov. Written informed consent was obtained from the surrogate of each participating patient, ensuring adherence to ethical standards and patient autonomy.

Patients admitted to the medical intensive care unit who were diagnosed with pneumonia underwent meticulous screening. The inclusion criteria were adult patients aged >21 years who had been intubated and received mechanical ventilation support for >48 h. Additionally, sputum cultures were required to confirm the presence of carbapenem-resistant or multidrug-resistant gram-negative bacteria, with a prescribed regimen of colistin administered intravenously (IV) alone or combined with inhalation. Specific clinical and radiological criteria included new persistent radiological infiltrates and specific clinical signs such as fever, purulent tracheal aspirations, or elevated leukocyte hospital-acquired pneumonia. Exclusion criteria were patients with recurrent carbapenemuse, corroborated by the clinical suspicion of drug-resistant gram-negative bacterial pneumonia; a history of colistin treatment prior to ICU admission; immunocompromised status (defined by neutropenia with an absolute neutrophil count < 500 cells/µL) due to conditions such as chemotherapy within the past three months; or renal insufficiency (defined as a creatinine clearance < 30 mL/min).

Patients who received IV colistin (TTY Biopharm, Taipei, Taiwan) alone were designated as controls for therapeutic effect comparisons over those who received inhaled plus IV colistin (IV + inhalation). The IV + inhalation group was allocated to two subgroups, with delivery facilitated by either a JN or VMN positioned at the inlet of the heated humidifier. Nebulizer selection was based on a computer-generated randomization schedule for number randomization, and each number was separately sealed in an opaque envelope. The envelopes were randomly drawn by a research assistant who administered the study protocols. The sample size for the IV alone or IV + inhalation groups was predetermined based on a previous study, and it was determined that a sample size of 36 was necessary to achieve a significance level (α) of 0.05, with 80% statistical power [10].

The administration protocol for colistin was standardized across groups, with patients in the IV group receiving a loading dose followed by a maintenance dose and those in the IV + inhalation group receiving additional inhaled colistin. The initiation of colistin for management was based on the patient’s clinical signs, microbiological findings, and antibiotic sensitivity test results after the patient was diagnosed with VAP. Dosage and administration details were calibrated according to a previous report [33]. Inhaled colistin in the IV + inhalation was administered in 1 vial comprising 2 million international units with 66·8 mg of colistin methanesulfonate, which was dissolved in 4 mL of normal saline via either a JN or VMN twice a day according to the randomized table.

The trial’s primary efficacy endpoint was the evaluation of the Clinical Pulmonary Infection Scores (CPIS), which provided a comprehensive assessment of pneumonia severity and response to treatment. Secondary endpoints encompassed a range of clinical parameters, including the Sequential Organ Failure Assessment (SOFA) score, Acute Physiology and Chronic Health Evaluation II (APACHE) scores, Charlson index, ventilator days, length of hospital and ICU stay, survival rate, and incidence of nephrotoxicity and bronchospasm.

Pulmonary and cardiovascular adverse events, including bronchospasm and changes in heart rate or lung sounds, were recorded to assess the treatment safety and tolerability. Nephrotoxicity was carefully monitored using serum creatinine and creatinine clearance levels at specified time points, with predefined criteria for early termination of colistin therapy in case of nephrotoxicity development when serum creatinine increased by ≥1.5 times the baseline [28].

### 4.2. In Vitro Drug Delivery Testing

The in vitro drug delivery was determined using a meticulously designed experimental setup. An adult ventilator (Galileo Hamilton Medical Inc., Prague, Czechia,) meticulously delivered specific respiratory parameters to mimic physiological conditions: tidal volume, 500 mL; respiratory rate, 20 breaths per minute; inspiratory time set at 1.2 s, and positive end-expiratory pressure, 8 cmH_2_O. To ensure optimal humidity, heated humidification was utilized (MR850, Fisher & Paykel Healthcare, Auckland, New Zealand) at a temperature of 37 ± 1 °C. This was integrated with a 7.5 mm endotracheal tube connected to a lung model characterized by a compliance of 0.04 L/cmH_2_O and an airway resistance of 5 cmH_2_O/L/s (Michigan Instrument Inc., Kentwood, MI, USA).

The nebulizer placement was strategically determined, either at the inlet of the heated humidifier or 15 cm from the Y-piece in the inspiratory limb of the ventilator circuit, to assess its impact on drug delivery efficiency. Colistin in powder form, comprising 2 million international units with 66·8 mg of colistin methanesulfonate, was dissolved in 4 mL of 0.9% normal saline and administered via either a Jet Nebulizer (JN, Galemed Corp, Taipei, Taiwan) or a Vibrating Mesh Nebulizer (VMN, Aerogen Ltd., Galway, Ireland). Its electronic controller powered the VMN, whereas the JN was powered by oxygen at a flow rate of 8 L/min, adhering to the manufacturer’s specifications. Nebulization time was precisely recorded to ensure consistency across the experiments.

A drug collection process was employed to quantify the delivered colistin using a filter integrated into the experimental setup. The collected colistin was then assayed using a meticulously validated High-Performance Liquid Chromatography (HPLC) system, specifically Alliance 2695 equipped with a photodiode array detector (Waters Corp., Milford, MA, USA), using a C18 column with a particle size of 5 μm. The column oven was maintained at a constant temperature of 10 °C throughout the experiments.

For chromatographic analysis, a trifluoroacetic acid 0.05% (*v*/*v*) water solution (Sol A) and acetonitrile (Sol B) mixture served as the mobile phase in a gradient elution process. Colistin was detected at a wavelength of 210 nm, with an injection volume of 20 μL and a flow rate of 1.00 mL/min. The retention time for colistin was consistently observed to be 10 min, ensuring the reliability and reproducibility of the results.

To validate the accuracy and precision of the assay, solutions of colistin in water were prepared at various concentrations ranging from 0.1 to 10 mg/mL, utilizing the United States Pharmacopeia (USP) colistin methanesulfonate reference standard (Sigma-Aldrich Corp., Saint Louis, MO, USA). The linearity of the assay was meticulously assessed across this concentration range (R2 = 0.9999), meeting the stringent USP test specifications. The percentage recovery of colistin from the working standard solution was 99.9%, with a confidence interval of 95%, ensuring the reliability and robustness of the analytical method employed.

### 4.3. Statistical Analysis

Categorical variables were expressed as percentages and continuous variables were represented as median and 25–75% interquartile range (IQR) or mean ± standard deviation. The delivered drug dose and nebulization time were compared using the Kruskal–Wallis and Mann–Whitney U tests. Differences among the three groups regarding clinical characteristics, duration of mechanical ventilation, and length of stay were compared using the Kruskal–Wallis test, and the differences between the two nebulizer groups were compared using the Mann–Whitney U rank-sum test. To evaluate nephrotoxicity, the Friedman test was used to compare changes in BUN levels and creatinine clearance among the groups at the three checkpoints and two weeks after the discontinuation of colistin. Data were analyzed using the Statistical Package for the Social Sciences (version 23; IBM Inc., Armonk, NY, USA). Statistical significance was set at *p* < 0.05.

## 5. Conclusions

In conclusion, compared to colistin through IV alone, adjunct inhalation colistin improved the CPIS score, APACHE score, and CRP level after 7 days, with no significant difference in survival rates or oxygenation among the three groups. Inhalation of colistin was safe and did not cause nephrotoxicity or bronchospasm. Despite the high delivery dose observed in the in vitro model, the outcomes in the inhalation groups were comparable between the two types of nebulizers.

## Figures and Tables

**Table 1:** Demographics and clinical characteristics of patients at baseline.

Characteristics	IV (n = 28)	IV + VMN (n = 17)	IV + JN (n = 15)	*p*-Value *
Age (yrs)	69.4 (59.2–80.7)	71.9 (59.1–82.7)	63.8 (57.2–76.9)	0.59
Male (%)	10 (35.7)	14 (82.3)	11 (73.3)	0.43
Diagnosis at admission				
Infection, n (%)	12 (42.8)	9 (52.9)	8 (53.3)	0.80
Pulmonology, n (%)	3 (10.7)	3 (17.6)	2 (13.4)	0.88
Oncology, n (%)	4 (14.2)	2 (11.6)	3 (20)	0.72
Cardiology, n (%)	1 (3.5)	1 (5.8)	0	1.0
Immunology, n (%)	2 (14.2)	0	1 (6.7)	0.56
Nephrology, n (%)	6 (21.3)	2 (11.6)	1 (6.7)	0.05
Past history				
Cardiovascular disease, n (%)	7 (25)	2 (11.7)	1 (6.7)	0.64
Pulmonary, n (%)	8 (28.5)	3 (17.6)	1 (6.7)	0.05
Cancer, n (%)	7 (25)	8 (47.1)	8 (53.3)	0.22
CKD, n (%)	5 (17.8)	3 (17.6)	2 (13.4)	0.89
Immunosuppression, n (%)	1 (3.55)	1 (5.8)	3 (20)	0.30
Severity indices				
CPIS	6 (4–8)	5 (3–7)	7 (5–8)	0.18
APACHE II score	26.5 (19.3–30.8)	22 (16–25)	22.5 (18–25.8)	0.40
SOFA	9.5 (7–14)	6 (5–8)	7.5 (6–12)	0.05
Charlson index	6 (4–10)	3.5 (2.5–5.0)	3.5 (2.3–5)	0.25
CRP (mg/L)	120.7 (61.2–170.6)	78.0 (43.9–99.6)	98.1 (40.0–130.9)	0.06
WBC (10^3^/uL)	12.2 (6.8–15.3)	9.8 (8.0–13.7)	12.2 (8.9–17.2)	0.43
Cr. (mg/dL)	1.3 (0.49–2.8)	0.72 (0.38–2.4)	0.93 (0.32–2.2)	0.43
BUN (mg/dL)	10.6 (23.7–96.2)	31.3 (18.1–77.5)	28.3 (16.4–54.9)	0.26
PaO_2_/FiO_2_ ratio	197 (107–251)	181 (148–278)	179 (98–225)	0.13
AaDO_2_	300.1 (182.8–352.4)	211.0 (192.4–248.9)	223.5 (175.2–458.2)	0.46

Data are presented as median (IQR). * Comparisons among three groups were analyzed by Kruskal–Wallis test. Abbreviations: IV, intravenous; JN, jet nebulizer; VMN, vibrating mesh nebulizer; CKD, chronic kidney disease; CPIS, Clinical Pulmonary Infection Score; APACHE, Acute Physiology and Chronic Health Evaluation; SOFA, Sequential Organ Failure Assessment; CRP, C-reactive protein; WBC, white blood cell; Cr, creatinine; BUN, blood urea nitrogen; PaO_2_/FiO_2_ ratio, partial arterial pressure to inspiratory oxygen fraction ratio; AaDO_2_, the difference between alveolar and arterial oxygen pressures.

**Table 2:** Comparison of CPIS scoring items after the administration of colistin among three groups.

	7th Day					10th Day				
Parameters	IV (n = 28)	IV + VMN (n = 17)	IV + JN (n = 15)	*p*-Value *	*p*-Value ^†^	IV (n = 28)	IV + VMN (n = 17)	IV + JN (n = 15)	*p*-Value *	*p*-Value ^†^
CPIS score	6.0 (5.0–8.0)	4.0 (3.0–7.3)	5.5 (3.0–7.8)	0.03	0.21	5.0 (3.0–7.3)	4.0 (3.0–7)	4.0 (4.0–7.8)	0.55	0.45
Temperature (°C)	36.4 (35.5–37.1)	36. (35.9–37.1)	36.9 (35.9–37.15)	0.29	0.96	37.0 (36.3–34.2)	37.2 (36.5–37.3)	37 (36.6–37.2)	0.66	0.54
WBC (10^3^/uL)	13.2 (8.3–16.6)	10.9 (6.7–12.8)	10.1 (8.8–14.2)	0.59	0.9109	12.5 (9.3–20.0)	13 (8.1–15.6)	11.1 (9.3–14.1)	0.71	0.93
Secretions	1	1	1	0.13	0.20	1	1	1	0.85	0.65
PaO_2_/FiO_2_	1 (1–2)	1 (1–2)	1 (1–2)	0.79	0.58	1 (1–2)	1 (1–1)	1 (1–2)	0.18	0.12
CXR	0	0	0	0.47	0.43	0	0	0	0.57	0.54
Microbiology	1 (0–1)	1 (0–1)	1(1–1)	0.05	0.05	1 (0–1)	0 (0–1)	1 (0–1)	0.24	0.23

Data are presented as median (IQR). * Comparisons among three groups were analyzed by Kruskal–Wallis test; ^†^ comparisons between two inhalation groups were analyzed by Mann–Whitney U-test. Abbreviations: IV, intravenous; JN, jet nebulizer; VMN, vibrating mesh nebulizer; CPIS, Clinical Pulmonary Infection Score; CXR, chest X-ray.

**Table 3:** Comparison of secondary outcomes after the administration of colistin among three groups.

	7th Day					10th Day				
Parameters	IV (n = 28)	IV + VMN (n = 17)	IV + JN (n = 15)	*p*-Value *	*p*-Value ^†^	IV (n = 28)	IV + VMN (n = 17)	IV + JN (n = 15)	*p*-Value *	*p*-Value ^†^
SOFA	9 (6–13)	6 (5–7)	7 (5–10)	0.007	0.21	8.5 (5–12)	6 (3.3–7)	7 (4.5–10)	0.05	0.29
APACHE score	26 (23.5–31.7)	21 (17.0–26.0)	21 (15.3–26)	0.002	0.96	25.5 (17.3–29.3)	21 (16–25.8)	18 (15.5–23)	0.19	0.62
Charlson index	5 (4–9)	4 (3–5)	5 (2–5)	0.72	0.42	6 (4–9)	4 (3–5)	5 (2–5)	0.68	0.64
CRP (mg/L)	137.5 (35.7–173.3)	46.1 (24.6–160.1)	43.2 (23.9–108.9)	0.02	0.98	77.8 (40.6–106.1)	58.2 (30.6–93.3)	50.1 (20.2–109.4)	0.22	0.63
PaO_2_/FiO_2_ ratio	266 (151–372)	293 (185–362)	170 (156–219)	0.35	0.69	247 (142–391)	231 (176–327)	152 (126–289)	0.17	0.07
AaDO_2_ (mmHg)	230 (204–277)	249 (191–289)	254 (172–350)	0.57	0.87	207 (181–263)	257 (181–293)	208 (166–255)	0.60	0.62
Cr. (mg/dL)	1.44 (0.58–2.81)	0.64 (0.48–2.29)	0.83 (38.8–2.04)	0.19	0.86	1.67 (0.68–3.06)	0.5 (0.36–1.26)	0.99 (0.58–2.82)	0.06	0.10
BUN (mg/dL)	64.3 (20.7–127.7)	19.3 (13.4–23.9)	31.2 (15.6–53.4)	0.007	0.73	56.6 (13.0–96.4)	24 (13.4–46.6)	32.4 (17.8–49.7)	0.29	0.38

Data are presented as median (IQR). * Comparisons among three groups were analyzed by Kruskal–Wallis test; ^†^ comparisons among two inhalation groups were analyzed by Mann–Whitney U test. Abbreviations: IV, intravenous; JN, jet nebulizer; VMN, vibrating mesh nebulizer; APACHE, Acute Physiology and Chronic Health Evaluation; SOFA, Sequential Organ Failure Assessment; CRP, C-reactive protein; Cr, creatinine; PaO_2_/FiO_2_ ratio, partial arterial pressure to inspiratory oxygen fraction ratio; AaDO_2_, difference between alveolar arterial oxygen pressure.

**Table 4:** Long-term clinical outcomes.

Parameters	IV (n = 28)	IV + VMN (n = 17)	IV + JN (n = 15)	*p*-Value *	*p*-Value ^†^
Ventilator days	24 (15.8–34.5)	37.5 (19.5–55.3)	27 (22–41)	0.17	0.14
Colistin days	12 (4.3–20.8)	11 (9–15)	11.5 (10–14.8)	0.79	0.69
ICU days	30.5 (22.5–49.7)	36 (27–80)	34.5 (27–78)	0.23	0.71
Hospital days	32 (25–77)	56 (41–98)	69 (54–77)	0.28	0.59
ICU survival rate (%)	67.9	89.5	62.5	0.06	0.18
Hospital survival rate (%)	53.6	84.2	50	0.06	0.09

Data are presented as median (IQR); * comparisons among three groups were analyzed by Kruskal-Wallis test; ^†^ comparisons among two inhalation groups were analyzed by Mann–Whitney U test. Abbreviations: IV, intravenous; JN, jet nebulizer; VMN, vibrating mesh nebulizer.

## Data Availability

The raw data supporting the conclusions of this article will be made available by the authors upon request.
